# Association between triglyceride-glucose index and thyroid function in euthyroid adults: The Korea National Health and Nutritional Examination Survey 2015

**DOI:** 10.1371/journal.pone.0254630

**Published:** 2021-07-15

**Authors:** Wonsuk Choi, Ji Yong Park, A. Ram Hong, Jee Hee Yoon, Hee Kyung Kim, Ho-Cheol Kang

**Affiliations:** Department of Internal Medicine, Chonnam National University Medical School, Gwangju, Republic of Korea; Boston University School of Medicine, UNITED STATES

## Abstract

**Objectives:**

Low-normal thyroid function is associated with numerous metabolic risk factors including insulin resistance (IR). Triglyceride-glucose (TyG) index is a new surrogate marker of IR calculated by fasting triglyceride and glucose levels. Here, we investigated the association between thyroid function and TyG index in non-diabetic euthyroid adults.

**Methods:**

This cross-sectional study was based on data from the Korean National Health and Nutritional Examination Survey 2015 including 1482 individuals (741 men and 741 women). Serum thyrotropin (TSH) and free thyroxine (fT4) levels were measured.

**Results:**

After adjusting for confounders, there was an inverse relationship of TyG index with fT4 in men (*β* = –0.094, *P =* 0.009) and a positive relationship of TyG index with TSH in women (*β* = 0.078, *P =* 0.018). The lowest fT4 tertile in men (*P =* 0.001) and the highest TSH tertile in women (*P =* 0.010) exhibited increased TyG index after adjusting for confounders. The lowest fT4 tertile also showed increased homeostatic model assessment for IR (HOMA-IR) only in men (*P =* 0.006). Odds ratios (ORs) for the high TyG index, which was defined as the highest TyG quartile decreased in the highest and second highest tertile of fT4 in men (OR = 0.41 and OR = 0.45, respectively; *P <* 0.001) and increased in highest tertile of TSH in women (OR = 1.81, *P =* 0.031) after adjusting for confounders. The OR for high HOMA-IR defined as the highest HOMA-IR quartile was also lower in the highest and second highest fT4 tertiles in men (both OR = 0.47; *P =* 0.003).

**Conclusions:**

This is the first study to suggest that TyG index is a good surrogate marker of IR in evaluating its relationship with thyroid function.

## Introduction

Thyroid hormone has pleiotropic functions in the regulation of multiple metabolic processes, including glucose metabolism, lipid metabolism, and energy expenditure [1]. Previous studies have uncovered associations between hypothyroidism and metabolic dysfunctions, such as type 2 diabetes mellitus (T2DM) and metabolic syndrome [2, 3]. Low-normal thyroid function, defined as high thyrotropin (TSH) and/or low free thyroxine (fT4) levels within the normal reference range, as well as subclinical and overt hypothyroidism, have been reported to be associated with metabolic syndrome [4–12]. Moreover, it has been demonstrated that low-normal thyroid function is associated with insulin resistance (IR), as indicated by homeostatic model assessment of IR (HOMA-IR) [11, 12].

The traditional use of HOMA-IR as a representative surrogate marker for IR is limited because fasting insulin levels are not frequently measured in real-world clinical settings [13]. To overcome this limitation, a new indicator for IR has been adopted recently: the triglyceride-glucose (TyG) index, which is calculated based on fasting triglyceride and glucose levels [14]. This index shows high sensitivity and specificity for identifying IR at various degrees of glucose tolerance and body weight as compared with the hyperinsulinemic-euglycemic clamp test, the standard method for evaluating IR [15]. Several studies have reported that a high TyG index is associated with incident cardio-metabolic diseases [16–18]. However, the association between thyroid function and the TyG index has not been studied to date.

Therefore, we here investigated the association between IR and thyroid function using the TyG index in non-diabetic, euthyroid Korean adults based on data from the Korean National Health and Nutritional Examination Survey (KNHANES) 2015.

## Materials and methods

### Patients

The KNHANES surveys were reviewed and approved by the Institutional Review Board of the Korea Disease Control and Prevention Agency (IRB No. 2015-01-02-6C). All participants provided informed consent prior to survey enrollment.

Since 1998, the Korea Disease Control and Prevention Agency has conducted the KNHANES, a cross-sectional nationwide survey representing the non-institutionalized civilian Korean population. The survey uses a stratified, multistage, and clustered probability sampling method based on age, sex, and geographical area.

We initially included 7,380 individuals who participated in the KNHANES 2015. Out of these, we excluded 5,898 subjects based on the following criteria: age <19 years (*n* = 1435); missing values for fasting plasma glucose (FPG), fasting triglycerides, fT4, or TSH (*n* = 3918); pregnant (*n* = 4); incident hyperthyroidism or hypothyroidism, including subclinical range of thyroid dysfunctions (*n* = 194); known thyroid diseases defined as previously diagnosed by a doctor or receiving any kind of treatments including levothyroxine and anti-thyroid drugs (*n* = 30); diabetes mellitus defined as taking anti-diabetic medications or glycated hemoglobin (HbA1c) ≥6.5% (*n* = 168); dyslipidemia defined as previously diagnosed by a doctor or taking any kind of lipid-lowering drugs (*n* = 112); cardiovascular diseases (*n* = 10); cerebrovascular diseases (*n* = 7); malignancy (*n* = 12); and chronic liver diseases (*n* = 8). Finally, 1,482 participants were included in the analyses.

The KNHANES surveys were reviewed and approved by the Institutional Review Board of the Korea Disease Control and Prevention Agency (IRB No. 2015-01-02-6C). All participants provided informed consent prior to survey enrollment.

### Demographic, lifestyle, and anthropometric parameters

Household interviews and physical examinations were conducted to collect data on age, sex, menopausal status for women, and health behaviors, including smoking history, alcohol consumption, and physical activity (PA). Smoking status was categorized as non-smoker, ex-smoker, or current smoker; the latter was defined as smoking at least five cigarettes daily during the previous 12 months. Alcohol consumption was divided into none, ex-drinker, and current drinker; the latter was defined as drinking alcohol at least once every month during the previous 12 months. PA was determined using the average metabolic equivalent of task (MET) score, which accounts for all types of exercise using the compendium of physical activities [19]. Walking, moderate PA, and vigorous PA were equivalent to MET scores of 3.3, 4.0, and 8.0, respectively. The total PA (MET-minute/week) was calculated as the sum of the weekly METs of the three PA categories. Body weight (kg) and height (cm) were measured using standard protocols, with the subjects dressed in light clothing without shoes. Body mass index (BMI) was calculated as body weight divided by the square of height in meters (kg/m^2^). Waist circumference was measured to the nearest 0.1 cm in a horizontal plane at the midpoint between the iliac crest and the costal margin at the end of a normal expiration.

### Laboratory assessment

All blood samples were obtained in the morning after fasting for at least 8 h. Serum TSH and fT4 levels were measured by electrochemiluminescence immunoassays (ECLIA) using an E-TSH kit (reference range 0.62–6.68 mIU/L) or an E-Free T4 kit (reference range 0.89–1.76 ng/dL), respectively (Roche Diagnostics, Mannheim, Germany) [20]. Serum insulin levels were measured by ECLIA using an E-Insulin kit (Roche Diagnostics, Mannheim, Germany). HbA1c levels were measured by high-performance liquid chromatography using a Tosoh G8 analyzer (Tosoh Bioscience, Tokyo, Japan). Serum total cholesterol, triglycerides, high-density lipoprotein (HDL) cholesterol, and FPG were measured using the Hitachi Automatic Analyzer 7600 (Hitachi, Tokyo, Japan). Serum low-density lipoprotein (LDL) cholesterol levels were calculated using the Friedewald formula [21]. Serum high-sensitivity C-reactive protein (hsCRP) levels were measured by immunoturbidimetry using Cobas 8000 (Roche Diagnostics, Mannheim, Germany). HOMA-IR was calculated using the formula FPG × fasting insulin/405 [13]. The TyG index was calculated using the following equation: ln (fasting triglycerides × FPG)/2 [22].

### Statistical analysis

Continuous and categorical variables are expressed as mean ± standard deviation or 95% confidence interval (CI) and as *n* (%), respectively. Between-group comparisons of clinical characteristics by sex were conducted using the Student’s t-test and Chi-square test. Pearson’s correlation coefficients were calculated to assess associations between TyG index and HOMA-IR. Because the TyG index and HOMA-IR were skewed, logarithmic transformations were performed for linear regression analyses. Adjusted multiple linear regression analysis was performed to evaluate the independent association of TSH or fT4 with TyG index and HOMA-IR after adjusting for age, BMI, waist circumference, smoking status, alcohol consumption, PA, menopause (in women), and hsCRP. Baseline characteristics according to the TSH and fT4 tertiles were compared using one-way analysis of variance (ANOVA) for continuous variables or Chi-square test for categorical variables. The Bonferroni correction was adopted as a post hoc analysis to account for multiple testing issues. To further analyze changes in TyG index and HOMA-IR according to the TSH or fT4 tertiles, multivariable-adjusted least square (LS) means with 95% CI were estimated and compared via analysis of covariance (ANCOVA) after adjusting for confounding factors. Odds ratios (ORs) and 95% CIs for high TyG index and HOMA-IR were compared using multiple logistic regression analyses. High TyG index and HOMA-IR were defined as the highest quartile of TyG index and HOMA-IR in each sex. All statistical analyses were performed using SPSS statistics for Windows (Version 25, IBM Corp., Armonk, NY, USA), and a *P* value of < 0.05 was considered as statistically significant.

## Results

### Baseline characteristics of the study population

The clinical characteristics of the study participants are summarized in Table 1. Mean age of men and women was 42.0 ± 14.7 and 41.3 ± 14.6 years, respectively (*P =* 0.334). BMI, waist circumference, and proportion of current smokers and alcohol drinkers were significantly higher in men than in women (all *P <* 0.001). Levels of fT4, FPG, insulin, HbA1c, triglycerides, LDL cholesterol, BUN, creatinine, AST, ALT, TyG index, and HOMA-IR were significantly higher in men than in women (*P <* 0.001 to 0.037), whereas TSH and HDL cholesterol levels were higher in women than in men (*P =* 0.018 and *P <* 0.001, respectively). There were no sex differences in the degree of PA, total cholesterol, and hsCRP.

**Table 1:** Baseline characteristics of the study subjects.

	Men (*n* = 741)	Women (*n* = 741)	*P*
Age (years)	42.0 ± 14.7	41.3 ± 14.6	0.334
BMI (kg/m^2^)	24.5 ± 3.5	22.9 ± 3.5	<0.001
Waist circumference (cm)	85.8 ± 9.2	77.2 ± 8.9	<0.001
Current smoker	279 (37.7)	41 (5.5)	<0.001
Current alcohol drinker	555 (74.9)	353 (47.6)	<0.001
PA (≥ MET 600 minute/week)	113 (15.2)	98 (13.2)	0.265
TSH (mIU/L)	2.38 ± 1.17	2.53 ± 1.24	0.018
fT4 (ng/dL)	1.29 ± 0.16	1.21 ± 0.14	<0.001
Fating plasma glucose (mg/dL)	96.0 ± 10.2	91.9 ± 8.7	<0.001
Insulin (mIU/L)	8.6 ± 7.5	7.3 ± 5.1	<0.001
HbA1c (%)	5.42 ± 0.32	5.39 ± 0.32	0.037
Total cholesterol (mg/dL)	191.8 ± 33.7	190.3 ± 33.4	0.379
Triglycerides (mg/dL)	159.5 ± 127.8	98.3 ± 59.0	<0.001
HDL cholesterol (mg/dL)	47.9 ± 11.3	55.9 ± 12.8	<0.001
LDL cholesterol (mg/dL)	117.2 ± 30.8	113.8 ± 30.6	0.030
BUN (mg/dL)	14.4 ± 3.6	12.9 ± 3.7	<0.001
Creatinine (mg/dL)	0.96 ± 0.12	0.71 ± 0.11	<0.001
AST (U/L)	24.3 ± 12.7	19.3 ± 6.8	<0.001
ALT (U/L)	27.1 ± 24.9	15.5 ± 10.2	<0.001
hsCRP (mg/L)	1.13 ± 1.95	1.19 ± 2.52	0.642
TyG index	4.71 ± 0.32	4.48 ± 0.27	<0.001
HOMA-IR	2.10 ± 2.12	1.70 ± 1.28	<0.001

Values are presented as mean ± standard deviation or *n* (%).

BMI, body mass index; PA, physical activity; MET, metabolic equivalent of task; TSH, thyrotropin; fT4, free thyroxine; HbA1c, glycated hemoglobin; HDL cholesterol, high-density lipoprotein cholesterol; LDL cholesterol, low-density lipoprotein cholesterol; BUN, blood urea nitrogen; AST, aspartate aminotransferase; ALT, alanine aminotransferase; hsCRP, high-sensitivity C-reactive protein; TyG, triglyceride-glucose index; HOMA-IR, homeostatic model assessment for insulin resistance.

### Associations of TyG index and HOMA-IR with TSH or fT4 in both sexes

The TyG index and HOMA-IR showed significant positive associations in men (*r* = 0.311, *P <* 0.001) and women (*r* = 0.381, *P <* 0.001). In men, the TyG index was negatively associated with fT4 in unadjusted and adjusted models (*β* = –0.187, *P <* 0.001 and *β* = –0.094, *P =* 0.009, respectively), whereas it was not associated with TSH in either model (Table 2). In women, the TyG index was positively associated with TSH in unadjusted and adjusted models (*β* = 0.079, *P =* 0.031 and *β* = 0.078, *P =* 0.018, respectively). The unadjusted model revealed a negative association between TyG index and fT4 in women (*r* = –0.116, *P =* 0.002), however, the statistical significance disappeared after adjusting for confounders (*P =* 0.163). HOMA-IR did not show significant associations with TSH or fT4 in either unadjusted or adjusted models in either sex.

**Table 2:** Associations of thyrotropin and free thyroxine levels with TyG index and HOMA-IR.

**Men**						
	Unadjusted			Adjusted^a^		
	*β*	SE	*P*	*β*	SE	*P*
(a) TyG index						
TSH	0.001	0.001	0.968	0.015	0.001	0.665
fT4	–0.187	0.006	<0.001	–0.094	0.006	0.009
(b) HOMA-IR						
TSH	0.054	0.010	0.139	0.028	0.008	0.360
fT4	–0.039	0.068	0.293	–0.026	0.060	0.428
**Women**						
	Unadjusted			Adjusted^a^		
	*β*	SE	*P*	*β*	SE	*P*
(a) TyG index						
TSH	0.079	0.001	0.031	0.078	0.001	0.018
fT4	–0.116	0.007	0.002	–0.047	0.006	0.163
(b) HOMA-IR						
TSH	0.016	0.008	0.655	0.034	0.007	0.288
fT4	–0.042	0.066	0.249	–0.008	0.059	0.800

SE, standard error.

^a^*P* values were generated by multiple linear regression analysis after adjusting age, BMI, waist circumference, smoking status, alcohol consumption, PA (≥ MET 600 minute/week), menopause (in women), and hsCRP.

### TyG index and HOMA-IR in relation to TSH and fT4 tertiles

We compared the characteristics of study participants according to tertiles of TSH (Table 3). In men, the proportion of current smokers (*P <* 0.001) and fT4 levels (*P =* 0.047) decreased with increasing TSH tertiles, whereas serum creatinine increased (*P <* 0.001). TyG index and HOMA-IR did not differ between TSH tertiles in men. In women, the proportion of current smokers (*P =* 0.025) and fT4 levels (*P =* 0.013) decreased with increasing TSH tertiles, whereas the level of TyG index increased (*P =* 0.016). There were no differences in HOMA-IR between TSH tertiles in women.

**Table 3:** Baseline characteristics according to TSH tertiles by sex.

	T1	T2	T3	*P*^a^
**(a) Men**	*n* = 246	*n* = 248	*n* = 247	
TSH range (mIU/L)	0.62–1.71	1.72–2.63	2.64–6.50	
Age (years)	42.4 ± 14.3	41.6 ± 14.7	42.1 ± 15.0	0.841
BMI (kg/m^2^)	24.3 ± 3.2	24.5 ± 3.4	24.6 ± 3.8	0.535
Waist circumference (cm)	85.4 ± 8.5	86.2 ± 8.9	85.9 ± 10.1	0.641
Current smoker	121 (49.2)	95 (38.3)	63 (25.5)	<0.001^b^
Current alcohol drinker	190 (77.2)	191 (77.0)	174 (70.4)	0.141^b^
PA (≥ MET 600 minute/week)	32 (13.0)	38 (15.3)	43 (17.4)	0.397^b^
fT4 (ng/dL)	1.30 ± 0.16	1.31 ± 0.16	1.27 ± 0.17^d^	0.047
Fasting plasma glucose (mg/dL)	95.9 ± 10.1	95.8 ± 10.3	96.2 ± 10.2	0.937
Insulin (mIU/L)	8.0 ± 7.7	8.8 ± 7.7	8.9 ± 7.2	0.367
HbA1c (%)	5.41 ± 0.32	5.43 ± 0.31	5.43 ± 0.33	0.790
Total cholesterol (mg/dL)	188.5 ± 33.5	192.2 ± 33.7	194.8 ± 33.7	0.109
Triglycerides (mg/dL)	148.9 ± 108.6	168.4 ± 150.4	161.3 ± 120.5	0.231
HDL cholesterol (mg/dL)	47.5 ± 11.7	48.2 ± 10.7	48.1 ± 11.5	0.785
LDL cholesterol (mg/dL)	115.3 ± 30.7	116.4 ± 29.7	119.9 ± 31.9	0.222
BUN (mg/dL)	14.1 ± 3.8	14.4 ± 3.5	14.6 ± 3.5	0.384
Creatinine (mg/dL)	0.93 ± 0.12	0.97 ± 0.12^c^	0.98 ± 0.12^c^	<0.001
AST (U/L)	23.4 ± 9.9	25.5 ± 15.3	23.9 ± 12.3	0.151
ALT (U/L)	24.6 ± 18.1	29.3 ± 30.2	27.3 ± 24.8	0.107
hsCRP (mg/L)	1.31 ± 2.55	1.00 ± 1.37	1.09 ± 1.75	0.199
TyG index	4.69 ± 0.31	4.72 ± 0.34	4.73 ± 0.31	0.363
HOMA-IR	1.99 ± 2.40	2.12 ± 2.02	2.17 ± 1.92	0.607
**(b) Women**	*n* = 249	*n* = 245	*n* = 247	
TSH range (mIU/L)	0.63–1.82	1.83–2.78	2.80–6.43	
Age (years)	40.1 ± 14.0	41.9 ± 14.8	41.9 ± 15.0	0.280
BMI (kg/m^2^)	22.9 ± 3.7	22.9 ± 3.3	22.8 ± 3.4	0.922
Waist circumference (cm)	77.1 ± 9.2	77.4 ± 8.6	77.1 ± 8.9	0.915
Current smoker	21 (8.4)	7 (2.9)	13 (5.3)	0.025^b^
Current alcohol drinker	123 (49.4)	117 (47.8)	113 (45.7)	0.718^b^
PA (≥ MET 600 minute/week)	31 (12.4)	36 (14.7)	31 (12.6)	0.709^b^
fT4 (ng/dL)	1.23 ± 0.15	1.22 ± 0.14	1.19 ± 0.14^d^	0.013
Fasting plasma glucose (mg/dL)	91.8 ± 8.8	91.8 ± 8.7	92.0 ± 8.5	0.981
Insulin (mIU/L)	7.6 ± 5.6	6.9 ± 4.0	7.5 ± 5.3	0.267
HbA1c (%)	5.38 ± 0.33	5.38 ± 0.30	5.40 ± 0.32	0.702
Total cholesterol (mg/dL)	188.2 ± 33.4	191.2 ± 33.2	191.5 ± 33.6	0.484
Triglycerides (mg/dL)	93.5 ± 53.1	96.3 ± 65.1	105.2 ± 57.7	0.069
HDL cholesterol (mg/dL)	55.9 ± 13.1	56.3 ± 12.7	55.4 ± 12.7	0.720
LDL cholesterol (mg/dL)	112.5 ± 30.5	114.3 ± 30.7	114.5 ± 30.7	0.721
BUN (mg/dL)	12.8 ± 3.7	12.9 ± 3.7	12.9 ± 3.6	0.950
Creatinine (mg/dL)	0.71 ± 0.12	0.72 ± 0.11	0.71 ± 0.09	0.231
AST (U/L)	19.2 ± 7.7	19.3 ± 6.0	19.5 ± 6.4	0.878
ALT (U/L)	15.6 ± 11.4	15.2 ± 8.5	15.7 ± 10.4	0.829
hsCRP (mg/L)	1.3 ± 2.8	1.2 ± 2.5	1.1 ± 2.2	0.665
TyG index	4.46 ± 0.26	4.46 ± 0.29	4.52 ± 0.26^c^^,^^d^	0.016
HOMA-IR	1.76 ± 1.40	1.59 ± 1.01	1.74 ± 1.40	0.285

Values are presented as mean ± standard deviation or *n* (%). T1, the first tertile; T2, the second tertile; T3, the third tertile.

^a^*P* values for these trends were generated by ANOVA.

^b^*P* values for these trends were generated by chi-square test.

^c^*P*<0.05 vs. T1 in the post-hoc analysis

^d^*P*<0.05 vs. T2 in the post-hoc analysis

We further examined the characteristics of the study participants with regard to the tertiles of fT4 (Table 4). In both sexes, age, BMI, waist circumference, FPG, HbA1c, triglycerides, AST, and TyG index decreased with increasing fT4 tertiles (*P <* 0.001 to 0.048). In men, total cholesterol, BUN, hsCRP, and HOMA-IR decreased with increasing fT4 tertiles (*P =* 0.001 to 0.013), whereas serum creatinine increased (*P =* 0.023). In women, HDL cholesterol increased with increasing fT4 tertiles (*P =* 0.006), whereas TSH and LDL cholesterol decreased (*P =* 0.044 and *P =* 0.020, respectively). HOMA-IR did not differ according to fT4 tertiles among women.

**Table 4:** Baseline characteristics according to fT4 tertiles by sex.

	T1	T2	T3	*P*^a^
**(a) Men**	*n* = 253	*n* = 233	*n* = 255	
fT4 range (ng/dL)	0.90–1.21	1.22–1.35	1.36–1.75	
Age (years)	47.5 ± 13.8	41.1 ± 14.3^c^	37.4 ± 14.0^c^^,d^	<0.001
BMI (kg/m^2^)	25.0 ± 3.5	24.4 ± 3.4	24.0 ± 3.4^c^	0.003
Waist circumference (cm)	87.4 ± 8.9	85.7 ± 9.3	84.4 ± 9.2^c^	0.001
Current smoker	84 (30.1)	102 (36.6)	93 (36.5)	0.050^b^
Current alcohol drinker	183 (72.3)	187 (80.3)	185 (72.5)	0.074^b^
PA (≥ MET 600 minute/week)	31 (12.3)	38 (16.3)	44 (17.3)	0.252^b^
TSH (mIU/L)	2.50 ± 1.24	2.28 ± 1.07	2.35 ± 1.18	0.114
Fasting plasma glucose (mg/dL)	98.6 ± 11.1	94.4 ± 9.3^c^	94.8 ± 9.5^c^	<0.001
Insulin (mIU/L)	9.6 ± 9.5	8.2 ± 7.0	7.9 ± 5.4^c^	0.020
HbA1c (%)	5.49 ± 0.33	5.42 ± 0.30^c^	5.36 ± 0.31^c^	<0.001
Total cholesterol (mg/dL)	197.7 ± 35.2	189.9 ± 32.2^c^	187.7 ± 32.9^c^	0.002
Triglycerides (mg/dL)	190.5 ± 158.7	150.3 ± 106.4^c^	137.3 ± 103.7^c^	<0.001
HDL cholesterol (mg/dL)	46.6 ± 11.4	48.5 ± 11.0	48.7 ± 11.4	0.083
LDL cholesterol (mg/dL)	119.7 ± 32.6	116.2 ± 28.5	115.7 ± 31.0	0.297
BUN (mg/dL)	14.9 ± 3.7	14.1 ± 3.6^c^	14.1 ± 3.4^c^	0.013
Creatinine (mg/dL)	0.95 ± 0.13	0.96 ± 0.12	0.98 ± 0.12^c^	0.023
AST (U/L)	26.8 ± 17.2	23.9 ± 10.2^c^	22.1 ± 8.4^c^	<0.001
ALT (U/L)	29.3 ± 28.8	26.6 ± 23.0	25.3 ± 22.2	0.174
hsCRP (mg/L)	1.47 ± 2.51	1.13 ± 2.03	0.80 ± 0.94^c^	0.001
TyG index	4.80 ± 0.34	4.69 ± 0.30^c^	4.64 ± 0.30^c^	<0.001
HOMA-IR	2.44 ± 2.80	1.96 ± 1.88^c^	1.88 ± 1.39^c^	0.006
**(b) Women**	*n* = 246	*n* = 248	*n* = 247	
fT4 range (ng/dL)	0.90–1.14	1.15–1.26	1.27–1.72	
Age (years)	44.0 ± 13.9	41.4 ± 15.3	38.5 ± 14.1^c^	<0.001
BMI (kg/m^2^)	23.3 ± 3.6	23.0 ± 3.5	22.3 ± 3.2^c^	0.004
Waist circumference (cm)	78.3 ± 8.9	77.5 ± 9.1	75.6 ± 8.6^c^	0.002
Current smoker	16 (6.5)	14 (5.6)	11 (4.5)	0.606^b^
Current alcohol drinker	126 (51.2)	108 (43.5)	119 (48.2)	0.228^b^
PA (≥ MET 600 minute/week)	38 (15.4)	28 (11.4)	32 (13.0)	0.390^b^
TSH (mIU/L)	2.68 ± 1.31	2.49 ± 1.19	2.41 ± 1.20^c^	0.044
Fasting plasma glucose (mg/dL)	93.0 ± 9.2	91.9 ± 9.1	90.8 ± 7.5^c^	0.014
Insulin (mIU/L)	7.6 ± 5.4	7.3 ± 4.6	7.2 ± 5.2	0.643
HbA1c (%)	5.45 ± 0.34	5.37 ± 0.30^c^	5.35 ± 0.31^c^	<0.001
Total cholesterol (mg/dL)	192.8 ± 34.4	191.7 ± 31.9	186.5 ± 33.5	0.080
Triglycerides (mg/dL)	105.1 ± 64.2	97.8 ± 54.0	92.1 ± 57.8^c^	0.049
HDL cholesterol (mg/dL)	54.8 ± 13.4	54.9 ± 11.5	58.0 ± 13.2^c^^,^^d^	0.006
LDL cholesterol (mg/dL)	115.8 ± 30.9	116.2 ± 29.6	109.3 ± 31.0^d^	0.020
BUN (mg/dL)	13.1 ± 3.8	13.1 ± 3.8	12.4 ± 3.4	0.063
Creatinine (mg/dL)	0.71 ± 0.12	0.71 ± 0.10	0.72 ± 0.10	0.285
AST (U/L)	20.1 ± 7.6	19.3 ± 6.7	18.6 ± 5.8^c^	0.048
ALT (U/L)	16.0 ± 11.2	15.8 ± 10.8	14.6 ± 8.2	0.275
hsCRP (mg/L)	1.34 ± 3.22	1.17 ± 2.09	1.04 ± 2.10	0.419
TyG index	4.52 ± 0.29	4.48 ± 0.27	4.45 ± 0.25^c^	0.032
HOMA-IR	1.78 ± 1.34	1.68 ± 1.17	1.63 ± 1.33	0.450

Values are presented as mean ± standard deviation or *n* (%). T1, the first tertile; T2, the second tertile; T3, the third tertile.

^a^*P* values for these trends were generated by ANOVA.

^b^*P* values for these trends were generated by chi-square test.

^c^*P*<0.05 vs. T1 in the post-hoc analysis

^d^*P*<0.05 vs. T2 in the post-hoc analysis

**Fig 1:**
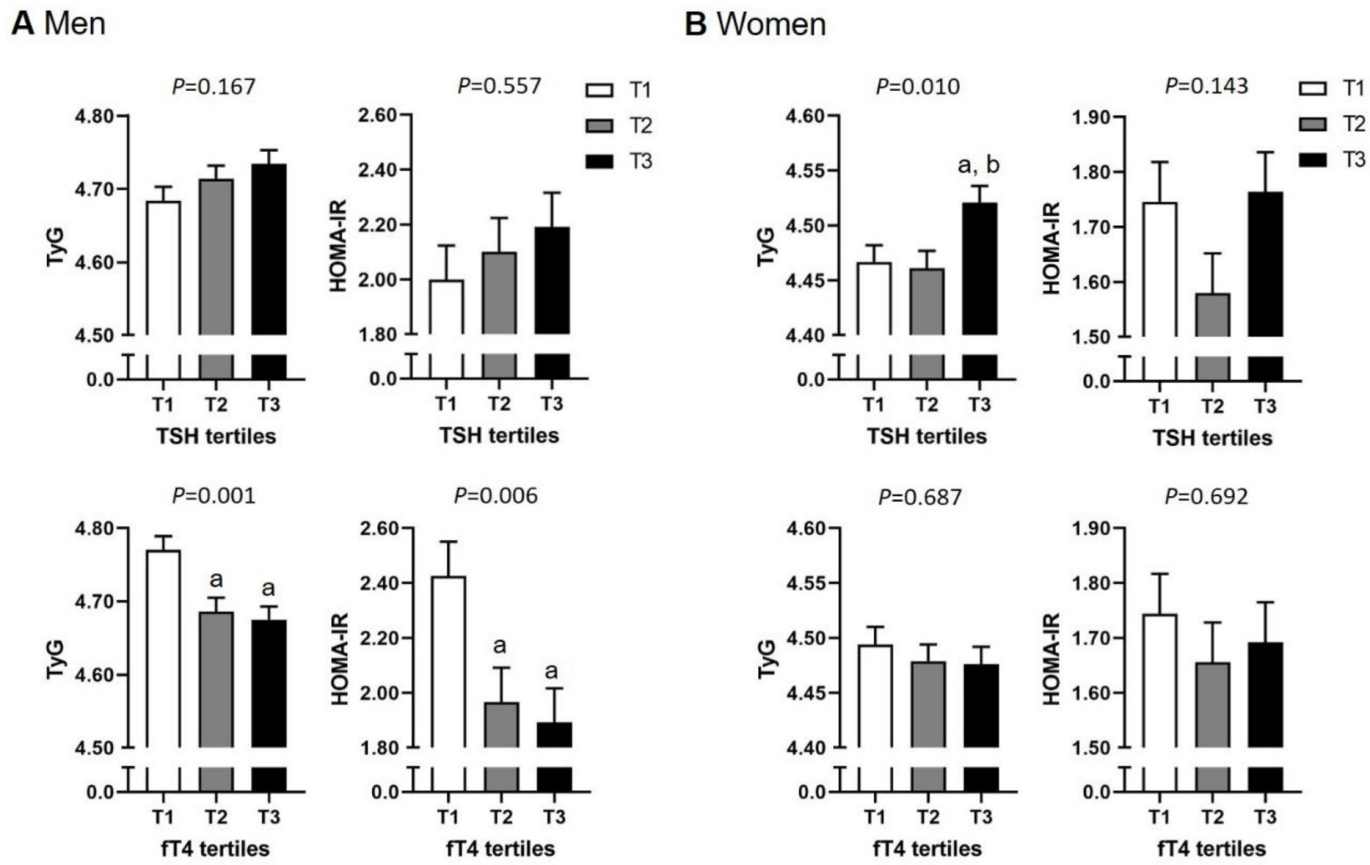
TyG index and HOMA-IR in (A) men and (B) women according to TSH tertiles and fT4 tertiles. Data are adjusted for confounders including age, BMI, waist circumference, smoking status, alcohol consumption, physical activity (≥MET 600 minute/week), menopause (in women), and hsCRP. Data are expressed as least square mean ± standard error. The *P* values for trends were generated by analysis of covariance (ANCOVA). ^a^*P <* 0.05 vs. the lowest tertile (T1) by ANCOVA. ^b^*P <* 0.05 vs. the second lowest tertile (T2) by ANCOVA.

Next, we compared the IR surrogate markers, TyG index and HOMA-IR, relative to TSH and fT4 tertiles after adjusting for confounding factors, including age, BMI, waist circumference, smoking status, alcohol consumption, degree of PA, menopause (in women), and hsCRP (Fig 1). In men, subjects in the lowest fT4 tertile exhibited the highest TyG index (LS mean difference between T1 and T2 of 0.084 [95% CI 0.019 to 0.148], LS mean difference between T1 and T3 of 0.095 [95% CI 0.030 to 0.160], *P =* 0.001), and HOMA-IR (LS mean difference between T1 and T2 of 0.461 [95% CI 0.032 to 0.890], LS mean difference between T1 and T3 of 0.535 [95% CI 0.102 to 0.968], *P =* 0.006) after adjusting for confounding factors. TyG index and HOMA-IR did not differ with respect to the TSH tertiles in men. In women, subjects in the highest TSH tertile exhibited the highest TyG index (LS mean difference between T1 and T3 of 0.054 [95% CI 0.002 to 0.107], LS mean difference between T2 and T3 of 0.060 [95% CI 0.008 to 0.113], *P =* 0.010) after adjusting for confounding factors, whereas there were no differences in HOMA-IR between TSH tertiles. TyG index and HOMA-IR did not differ according to fT4 tertiles in women.

### Risk of high TyG index and HOMA-IR regarding TSH and fT4 tertiles

Finally, we investigated the risk of high TyG index and HOMA-IR, which were defined as the highest quartiles in each sex, according to TSH and fT4 tertiles. After adjusting for all confounding factors, ORs for high TyG index were significantly lower in the highest and second highest fT4 tertiles in men (OR = 0.41, 95% CI 0.26 to 0.64 and OR = 0.45, 95% CI 0.29 to 0.69, respectively; *P <* 0.001), whereas it was significantly higher in the highest TSH tertile in women (OR = 1.81, 95% CI 1.15 to 2.84, *P =* 0.031) (Table 5). The OR for high HOMA-IR was significantly lower in the highest and second highest fT4 tertiles in men (OR = 0.47, 95% CI 0.29 to 0.78 and OR = 0.47, 95% CI 0.28 to 0.78, respectively; *P* = 0.003), but not in women (*P* = 0.476). There were no differences in the risk of high HOMA-IR with respect to TSH tertiles in men and women (*P =* 0.207 and *P =* 0.297, respectively).

**Table 5:** Odds ratio for high TyG index and HOMA-IR with respect to TSH or fT4 tertiles by sex.

	Men		Women	
	OR (95% CI)	*P*^a^	OR (95% CI)	*P*^a^
(a) High TyG index				
TSH tertiles		0.286		0.031
T1	Ref		Ref	
T2	1.16 (0.75–1.81)		1.25 (0.79–1.99)	
T3	1.43 (0.92–2.24)		1.81 (1.15–2.84)	
fT4 tertiles		<0.001		0.202
T1	Ref		Ref	
T2	0.45 (0.29–0.69)		0.73 (0.48–1.13)	
T3	0.41 (0.26–0.64)		0.69 (0.44–1.08)	
(b) High HOMA-IR				
TSH tertiles		0.207		0.297
T1	Ref		Ref	
T2	1.39 (0.85–2.27)		1.18 (0.74–1.88)	
T3	1.55 (0.94–2.56)		1.44 (0.91–2.29)	
fT4 tertiles				
T1	Ref	0.003	Ref	0.476
T2	0.47 (0.28–0.78)		0.77 (0.49–1.22)	
T3	0.47 (0.29–0.78)		0.79 (0.50–1.26)	

^a^*P* was generated by multiple logistic regression analysis after adjusting age, BMI, waist circumference, current smoker, current alcohol drinker, physical activity (≥MET 600 minute/week), menopause (in women), and hsCRP. High TyG and HOMA-IR were defined as each values in the highest quartile for both sex.

## Discussion

The present study demonstrates that in a representative cohort of the Korean population, based on data from the KNHANES 2015, the TyG index was associated with low-normal thyroid function in non-diabetic, euthyroid adults. While it was negatively associated with fT4 in men, it was positively associated with TSH in women. These observations remained statistically significant after adjusting for confounding factors. Furthermore, the lowest fT4 tertile in men and the highest TSH tertile in women were significantly associated with the risk of a high TyG index.

The TyG index, which is a product of fasting triglycerides and glucose, is a recently proposed surrogate marker of IR [14, 15]. Not only is it more cost-effective in a real-world clinical setting, it has also been reported to outperform HOMA-IR in the prediction of incident cardio-metabolic diseases, such as T2DM, non-alcoholic fatty liver disease (NAFLD), and cardiovascular diseases [16–18, 23–25]. To the best of our knowledge, this is the first study to examine the association between the TyG index and thyroid function.

We investigated this relationship according to sex in non-diabetic, euthyroid adults. Similar to previous studies [14, 26], the TyG index showed a positive correlation with HOMA-IR, a traditionally used surrogate marker of IR. In men, we observed a negative association between fT4 levels and the TyG index in both unadjusted and adjusted analyses. In contrast, women exhibited a positive association between the TyG index and TSH, regardless of adjustment. However, the negative correlation we found between fT4 concentrations and TyG index in women in our unadjusted analysis disappeared after adjusting for confounding factors. These findings indicate that although the components of thyroid function related to the TyG index differ between sexes, there is an independent association between the TyG index and low-normal thyroid function in both men and women.

We observed that male subjects in the lowest fT4 tertile had a higher TyG index compared with those in the higher fT4 tertiles after adjusting for confounding factors. In women, subjects in the highest TSH tertile had a higher TyG index compared with those in the lower TSH tertiles after adjusting for confounders. We further investigated the risk of IR, defined as the highest quartile of TyG index, with respect to TSH and fT4 tertiles. The lowest fT4 tertile in men and the highest TSH tertile in women showed a significantly increased risk of a high TyG index. Taken together, our findings suggest a strong association between low-normal thyroid function and IR in terms of the TyG index.

In order to compare the TyG index to traditional methodologies, we examined the association between HOMA-IR and thyroid function. Interestingly, HOMA-IR did not show significant associations with TSH or fT4 in either men or women using multiple linear regression analyses. However, in men, the lowest fT4 tertile showed a higher HOMA-IR compared with higher fT4 tertiles after adjusting for confounding factors. Similar to the TyG index, we further evaluated the risk of high HOMA-IR (defined as the highest quartile of HOMA-IR) with respect to TSH and fT4 tertiles. The lowest fT4 tertile in men correlated with an increased risk of high HOMA-IR, whereas no such relationships were observed in women. These findings differ from those of previous studies, which reported a significant association of both TSH and fT4 with HOMA-IR [11, 12]. The discrepancy between the current study and others may be due to several reasons. First, whilst ours was conducted on a Korean population, the subjects of aforementioned previous studies were Dutch [11] and Mexican [12]. Interestingly, two studies conducted on Koreans showed a significant association of TSH with metabolic syndrome, but not with HOMA-IR, in euthyroid women, suggesting possible ethnic differences in this context [4, 7]. Pancreatic beta cell dysfunction has a greater contribution to the development of type 2 diabetes in Asians compared to other ethnicities [27, 28]. Therefore, when the degree of metabolic dysfunction is similar, the serum insulin level in Asians is relatively low. This may be the reason for inconsistent results from studies of Koreans and other ethnic groups on the association of thyroid function with HOMA-IR. Second, we included only euthyroid subjects, in contrast to a previous study that included subjects with subclinical hypothyroidism, as well as euthyroidism [12]. Third, there are methodological differences in measuring TSH and fT4, and regarding the reference ranges across the studies. Finally, we analyzed men and women separately due to different baseline characteristics between sexes, whereas previous studies analyzed all study participants after adjusting for age and sex. These differences across studies might elicit discordant results for the association between thyroid function and HOMA-IR. Given our results, we nevertheless speculate that the TyG index could be a stronger indicator for assessing IR with respect to thyroid function, as compared with HOMA-IR. However, further studies will be needed to validate our findings, particularly the relative association between TyG index and HOMA-IR in the assessment of thyroid function.

The relationship between low-normal thyroid function and metabolic dysfunction has been previously reported. Some studies have shown that components of metabolic syndrome are associated with high TSH [4–7], while others have found associations with low fT4 [8–10] or both [11, 12]. In addition, low-normal thyroid function is associated with incident T2DM [29]. These findings may be explained by the direct biological effects of TSH and fT4 on glucose and lipid metabolism: TSH directly stimulates gluconeogenesis [30], cholesterol synthesis [31, 32], de novo lipogenesis in hepatocytes [33], and leptin secretion in adipocytes [34], while low fT4 directly causes decreased glucose utilization in skeletal muscles [35–37] and adipocytes [35, 37]. However, the relative contribution of TSH and fT4 to metabolic dysfunction has not been fully elucidated.

A recent study has reported that the association between mild elevation in TSH and lipid levels varies with menopausal status [38]. In premenopausal women, TSH showed a significant positive relationship with total cholesterol, LDL cholesterol, and triglycerides, whereas in postmenopausal women, TSH showed a significant positive relationship only with triglycerides. Because our study investigated the association between TSH and triglyceride-based TyG index, we adjusted for menopausal status as a covariate in the multivariate analyses. Therefore, our results are likely independent of women’s menopausal status.

This study has some limitations. First, due to the cross-sectional design of the study we could not determine the causality of low-normal thyroid function using the TyG index. Second, the classification of IR (indicated as high TyG index or HOMA-IR), defined as the highest quartile of TyG index and HOMA-IR in each sex, may be arbitrary. Although there are no definite cut-off values for IR and previous studies used a similar classification [26, 39–41], our results might be biased by the non-definite classification of IR. Further, in order to clearly confirm the usefulness of the TyG index, its associations with subclinical and overt thyroid dysfunction should be evaluated in future studies.

Taken together, this study shows that low-normal thyroid function was associated with a higher TyG index in euthyroid Korean adults. Particularly the relationship between the different components of low-normal thyroid function and the TyG index were found to differ according to sex, namely fT4 in men and TSH in women. Our findings suggest that the TyG index is a good surrogate marker of IR in evaluating its relationship with thyroid function.
